# Th1, Th2, and Th17 Cytokine Involvement in Thyroid Associated Ophthalmopathy

**DOI:** 10.1155/2015/609593

**Published:** 2015-05-18

**Authors:** Jie Shen, Zhangfang Li, Wenting Li, Ying Ge, Min Xie, Meng Lv, Yanfei Fan, Zhi Chen, Defu Zhao, Yajuan Han

**Affiliations:** ^1^Department of Endocrinology and Metabolism, The Third Affiliated Hospital of Southern Medical University, Guangzhou 510630, China; ^2^Department of Endocrinology and Metabolism, Guangzhou City People's Hospital, Huadu District, Guangzhou, China; ^3^Department of Endocrinology and Metabolism, Nanjing Jiangning Hospital, Nanjing, China; ^4^Department of Endocrinology and Metabolism, Guangzhou Military General Hospital, Guangzhou, China

## Abstract

To determine serum cytokine profiles in Graves' disease (GD) patients with or without active and inactive thyroid associated ophthalmopathy (TAO), we recruited 65 subjects: 10 GD only (without TAO), 25 GD + active TAO, 20 GD + TAO, and 10 healthy controls. Liquid chip assay was used to measure serum Th1/Th2/Th17 cytokines including IFN-*γ* (interferon-gamma), TNF-*α* (tumor necrosis factor-alpha), IL-1*α* (interleukin-1 alpha), IL-1Ra (IL-1 receptor antagonist), IL-2, IL-4, IL-6, and IL-17 and two chemokines: RANTES (regulated upon activation, normal T cell expressed and secreted) and IP-10 (IFN-*γ*-induced protein 10). Serum levels of TSH (thyroid stimulating hormone) receptor autoantibodies (TRAb) were measured using an enzyme linked immunosorbent assay. Compared with healthy controls, TAO patients showed significantly elevated serum levels of IFN-*γ*, TNF-*α*, IL-1*α*, IL-4, IL-6, IL-17, and IP-10. Comparing active and inactive TAO, serum Th1 cytokines IFN-*γ* and TNF-*α* were elevated in active TAO, while serum Th2 cytokine IL-4 was elevated in inactive TAO. Serum Th17 cytokine IL-17 was elevated in GD but reduced in both active and inactive TAO. A positive correlation was found between TRAb and IFN-*γ*, TNF-*α*, IL-1*α*, IL-2, IL-4, and IL-6. Taken together, serum Th1/Th2/Th17 cytokines and chemokines reflect TAO disease activity and may be implicated in TAO pathogenesis.

## 1. Introduction

Thyroid associated ophthalmopathy (TAO) is a thyroid-related eye autoimmune disorder characterized by diffuse goiter, thyrotoxicosis, and infiltrate orbitopathy [1, 2]. It is also called Graves' ophthalmopathy (GO) because at least 80% of patients with TAO also present with Graves' disease (GD). GD is an autoimmune disease which primarily affects the thyroid and causes the overproduction of thyroid hormones (hyperthyroidism). Hyperthyroidism leads to various symptoms which significantly influence patients' overall well-being, including increased heartbeat, muscle weakness, disturbed sleep, and irritability. Twenty-five to fifty percent of GD patients develop TAO [2–4]. The common clinical features of TAO are proptosis, upper eyelid retraction, edema, erythema of the periorbital tissues and conjunctivae, and visual field defects or vision loss, all of which greatly affect the quality of life of TAO patients. The natural course of TAO is an active (inflammatory) phase in the initial stage, subsequently followed by an inactive (fibrotic) phase in the late stage [5]. One-third of TAO patients may develop moderate or severe disease and need to be treated as promptly as possible during the active phase [4, 6]. Medical treatments are generally effective in patients with active TAO but are ineffective in cases of inactive TAO. Therefore, the early assessment of TAO disease activity is essential for optimal outcome of medical management.

Although it is commonly believed that multiple genetic, epigenetic, environmental, and immunological factors orchestrate the development of TAO, the pathogenesis of TAO is not fully understood [7]. Among the immunological factors, orbital fibroblasts and autoreactive lymphocytes play key roles in the initiation and propagation of TAO [8–10]. Insulin-like growth factor-1 receptor (IGF-1R) expressed on the cell surface of orbital fibroblasts might serve as an autoantigen to mediate activation of B and T cells [11–13]. Activated B cells produce autoantibodies and T cells secret cytokines which in turn stimulate orbital fibroblast proliferation and secretion of hydrophilic glycosaminoglycans [14, 15]. In addition, activation of IGF-1R also stimulates orbital fibroblasts to secrete chemokines for T cell recruitment [11, 13].

The roles of T cells in TAO have been established but an understanding of the underlying molecular interregulation and crosstalk are still lacking [16, 17]. Yang et al. found that the lymphocytes infiltrating in the retrobulbar space of TAO patients are primarily CD4+ T helper (Th) cells [18]. Due to the difficulty of accessing the retrobulbar space, serum has become a better choice for measuring systemic cytokines. Th-related cytokines are further divided into Th1 and Th2 cytokines [19]. Th1 cytokines, such as interleukin-2 (IL-2), interferon-*γ* (IFN-*γ*), tumor necrosis factor-*α* (TNF-*α*), and IL-12, primarily mediate cellular immunity while Th2 cytokines, such as IL-4, IL-5, IL-6, and IL-10, are involved in humoral immunity to mediate differentiation and antibody production of B cells [20]. Previous reports demonstrated that some Th1 and Th2 cytokines are elevated in serum of TAO patients [21, 22]. Moreover, a new subset of T helper cells, Th17, which primarily produce IL-17 has been found and might be involved in GD pathogenesis [23, 24]. IL-17 is a potent proinflammatory cytokine and only very recently has been shown to increase in serum of TAO patients [25, 26]. However, whether these Th1, Th2, and Th17 cytokines are involved in TAO disease activity is not yet clear.

To gain insight into the pathogenesis of TAO, this study used a bead-based multiplex sandwich immunoassay (Luminex) to detect serum Th1, Th2, and Th17 cytokines and chemokines in patients with GD without TAO, in patients with GD with active TAO, and in patients with GD with inactive TAO. This study used a multiplex Luminex assay which allowed us to simultaneously detect multiple cytokines/chemokines while conserving limited blood samples; sampling from the three distinct patient groups allowed us to observe kinetic changes in serum cytokines/chemokines during the development of TAO. Since one of the goals of TAO treatment is to inhibit the dysregulated autoimmune processes, our results might identify potential molecular targets for development of future TAO therapy.

## 2. Materials and Methods

### 2.1. Subjects

This study was approved by the institutional review board and informed consents were obtained before blood collection. Forty-five TAO cases with stable thyroid function and without treatment of high dose glucocorticoid pulse and other immunosuppressive agents were recruited between January 2011 and February 2012 in the Department of Endocrinology and Metabolism, the Third Affiliated Hospital of Southern Medical University, China. Clinical activity score (CAS) characterization was performed as described previously [27]. Twenty-five patients with GD and active TAO (CAS ≥ 3) and 20 patients with GD and inactive TAO (CAS < 3) were enrolled in this study. Ten patients with GD but without TAO and 10 healthy volunteers were also recruited. The 4 study groups will hereafter be referred to as GD, active TAO, inactive TAO, and control (normal) groups.

### 2.2. Assessment of Serum Cytokines and TRAb

Serum levels of cytokines and TSH (thyroid stimulating hormone) receptor autoantibodies (TRAb) were determined using a Milliplex MAP Human Cytokine panel I kit (Millipore, Germany) with LiquiChip Luminex (Qiagen, USA) and TRAb detection kits (RSR Ltd., UK), respectively, according to the manufacturer's instructions. The intra- and interassay coefficients of variations and sensitivity/mean minimum detectable concentration of each cytokine and chemokine were as follows: IFN-*γ* (1.6%, 12.0%; 0.8 pg/mL), TNF-*α* (2.6%, 13.0%; 0.7 pg/mL), IL-1*α* (3.3%, 12.8%; 9.4 pg/mL), IL-1Ra (2.1%, 10.7%; 8.3 pg/mL), IL-2 (2.1%, 6.3%; 1.0 pg/mL), IL-4 (2.9%, 14.2%; 4.5 pg/mL), IL-6 (2.0%, 18.3%; 0.9 pg/mL), IL-17 (2.2%, 7.9%; 0.7 pg/mL), IP-10 (2.6%, 15.3%; 8.6 pg/mL), and RANTES (1.9%, 5.0%; 1.2 pg/mL).

### 2.3. Statistical Analysis

Statistical analyses were conducted with SPSS 13.0 for Windows. All data are presented as means ± SD. Differences between groups were analyzed using one-way analysis of variance (ANOVA), and multiple comparisons were analyzed by the LSD method when *p* values were less than 0.05. The Welch method was used when equal variances were not assumed, and multiple comparisons were analyzed by the Dunnett T3 method when *p* values were less than 0.05. Statistical significance was accepted at a value of *p* < 0.05. The correlation between serum cytokines and TRAb was analyzed using Spearman correlation analysis.

## 3. Results

### 3.1. Demographic Data

Sixty-five subjects were recruited into this study: 10 normal controls (34.3 ± 12.2 years; 5 males and 5 females), 10 GD patients without TAO (32.8 ± 10.7 years; 5 males and 5 females), 20 patients with inactive TAO (CAS < 3; 38.3 ± 11.2 years; 11 males and 9 females), and 25 patients with active TAO (CAS > 3; 39.6 ± 15.1 years; 12 males and 13 females). The demographic features are shown in Table 1. There was no significant difference among the four groups regarding age (*F*/*χ* ^2^ = 0.883; *p* = 0.455), sex (*F*/*χ* ^2^ = 0.225; *p* = 0.974), and smoking habits (*F*/*χ* ^2^ = 1.308; *p* = 0.727).

### 3.2. T Helper 1 (Th1) Cytokines

To investigate the roles of serum cytokines in the pathogenesis of TAO, we first determined the levels of Th1 cytokines which have been shown to mediate the progression of various autoimmune diseases [28]. Using the LiquiChip Luminex system, we were able to detect multiple serum Th1 cytokines (including IFN-*γ*, TNF-*α*, IL-1*α*, IL-1Ra, and IL-2) simultaneously. Results are shown in Table 2. Among normal, GD, GD with inactive TAO, and GD with active TAO groups, there were significant differences in IFN-*γ* (*F* = 30.290; *p* = 0.000), TNF-*α* (*F* = 14.034; *p* = 0.000), IL-1*α* (*F* = 242.21; *p* = 0.000), and IL-2 (*F* = 279.1; *p* = 0.000), but not in IL-1Ra (*F* = 0.894; *p* = 0.450). When compared to the normal control, the GD only group showed significant elevation in TNF-*α* (*p* < 0.05), IL-1*α* (*p* < 0.01), and IL-2 (*p* < 0.01); the GD plus inactive TAO group showed significant elevation in IFN-*α* (*p* < 0.01), TNF-*α* (*p* < 0.05), and IL-1*α* (*p* < 0.01); and the GD plus active TAO group showed significant elevation in IFN-*γ* (*p* < 0.01), TNF-*α* (*p* < 0.01), and IL-1*α* (*p* < 0.01). When compared to the GD only group, both inactive and active TAO showed significant reduction in IL-2 (*p* < 0.01). When compared to the inactive TAO group, the active TAO group showed significant elevation in IFN-*γ* (*p* < 0.05) and TNF-*α* (*p* < 0.05). Taken together, serum TNF-*α* and IL-1*α* were elevated in both GD only group and GD plus TAO group, and IL-2 was decreased when TAO was developing. Regarding TAO activity, results suggest that serum IFN-*γ* and TNF-*α* might be important mediators for TAO pathogenesis as well as good markers to monitor active TAO.

### 3.3. T Helper 2 (Th2) Cytokines

To further explore the involvement of cytokines in TAO pathogenesis, we also examined the serum levels of Th2 cytokines including IL-4 and IL-6. Results are shown in Table 3. Among the four groups, there were significant differences in IL-4 (*F* = 117.00; *p* = 0.000) and IL-6 (*F* = 678.47; *p* = 0.000). When compared to the normal control, each group showed significant elevation in IL-4 (*p* < 0.01) and IL-6 (*p* < 0.01). When compared to GD only group, inactive TAO showed significant reduction in IL-6 (*p* < 0.01) and active TAO showed significant reduction in both IL-4 (*p* < 0.01) and IL-6 (*p* < 0.01). When compared to the inactive TAO group, active TAO group showed significant reduction in IL-4 (*p* < 0.01). Taken together, serum IL-4 and IL-6 increased in both the GD only group and the GD plus TAO group. Serum IL-4 was elevated in the inactive TAO group, but decreased when TAO progressed from an inactive to active phase.

### 3.4. T Helper 17 (Th17) Cytokine and Two Chemokines

Distinct from Th1 and Th2 cells, Th17 cells are another subset of T helper cells; they produce cytokine IL-17 [29, 30]. Because chemokines may be involved in recruiting immune cells to the eye tissue of TAO patients, we were interested in investigating their roles in TAO. Results are shown in Table 4. Among the four groups, there were significant differences in IL-17 (*F* = 66.206; *p* = 0.000) and IP-10 (*F* = 17.131; *p* = 0.000), but not in RANTES (*F* = 0.212; *p* = 0.887). When compared to the normal control, each group showed significantly higher levels of IL-17 and IP-10. When compared to the GD only group, both inactive and active TAO showed significant reduction in IL-17 (*p* < 0.01) and IP-10 (*p* < 0.01). We found slightly elevated levels of IL-17 and IP-10 in active TAO when compared to inactive TAO, but the difference was not statistically significant. These results suggest that serum IL-17 and IP-10 are elevated in GD but are reduced as TAO develops.

### 3.5. Correlation between Serum Cytokines and TRAb in TAO

Measuring TSH (thyroid stimulating hormone) receptor autoantibodies (TRAb) is the standard method for determining the disease activity of TAO. We therefore performed correlation analyses between serum levels of TRAb and the cytokines we measured in TAO groups. Results are shown in Table 5. The serum levels of TRAb were correlated with serum levels of IFN-*γ*, TNF-*α*, IL-1*α*, IL-2, IL-4, and IL-6 (*r* = 0.470, 0.309, 0.310, −0.352, −0.340, and 0.332; *p* = 0.001, 0.039, 0.038, 0.018, 0.022, and 0.026) but not with IL-1Ra, IL-17, RANTES, and IP-10 (*p* > 0.05). Results indicated a positive correlation of serum levels of IFN-*γ*, TNF-*α*, IL-1*α*, and IL-6 with TAO disease activity, while IL-2 and IL-4 negatively correlated with TAO disease activity.

## 4. Discussion

Given the important roles of cytokines in the immunopathogenesis in both GD and TAO [9, 14], we sought to examine a broad spectrum of serum cytokines in GD and TAO patients with different extents of disease activity. In the present study, we showed that serum levels of the Th1 cytokines IFN-*γ* and TNF-*α* were elevated in active TAO while serum Th2 cytokine IL-4 was elevated in inactive TAO. Serum cytokine IL-17 was elevated in the GD alone group but was reduced in both the active and the inactive TAO groups. These findings suggest that serum Th1/Th2/Th17 cytokines profiles might be useful as indicators for determining TAO development and disease activity. As this is a preliminary study, the sample size was small; a future investigation with a larger sample size is warranted.

Previous reports from other researchers have demonstrated that serum IL-6 and TNF-*α* are elevated in TAO patients, with the exception of IL-1Ra which did not correlate either with the parameters of GD or with TAO activity [22]. Two recent reports also demonstrated elevated serum IL-2 levels in GD patients as well as elevated IL17 levels in both GD and TAO patients [21, 25]. Our results are in agreement with previous reports and expand upon earlier studies; here, more cytokines, including IFN-*γ*, IL-1*α*, and IL-4, and a chemokine IP-10 were elevated in GD and TAO patients. To the best of our knowledge, this is the first study showing the possible role of Th1/Th2/Th17 cytokines in the disease activity of TAO. In addition, we found that some discrepancy regarding the cytokine and chemokine levels in the control groups was somewhat different from others' studies [31–36] with larger sample size. The levels of IL-1Ra, IL-2, IL-4, and RANTES in control group were lower than those in others' studies. The possible reasons for the discrepancy might be due to the differences in the sample size or detection method (multiplex Luminex assay versus traditional ELISA). Ethnicity (Chinese people versus Caucasians) or demographic data (sex and age) of the enrolled subjects might be possible reasons, but further investigation was needed to clarify the discrepancy.

IFN-*γ* and TNF-*α* are representative Th1 cytokines which have been implicated in TAO [37–40]. Wang et al. reported that IFN-*γ* stimulates retroorbital fibroblasts proliferation and the synthesis of hyaluronic acid [37]. IFN-*γ* and TNF-*α* may also modulate the expression of orbital autoantigen TSH receptor [39]. Using retrobulbar fibroblasts, retrobulbar preadipocytes from TAO patients, and primary cultures of thyrocytes, Antonelli et al. showed that IFN-*γ* and TNF-*α* treatment induced IP-10 release through a PPAR*γ*- (peroxisome proliferator-activated receptor-gamma-) dependent pathway [38, 40]. As shown in Table 2, we demonstrated a kinetic change of serum IFN-*γ* and TNF-*α* levels in GD, inactive TAO, and active TAO. The results were in disagreement with others' studies [41–43] reporting that the levels of IFN-*γ* and TNF-*α* had no differences among GD, TAO, or controls as well as no correlation among GD or TAO with TRAb levels. The aforementioned discrepancies could suggest the need to increase the number of studied patients and to compare the results with those obtained with other methods that should be used, too. But since previous reports have established the pathological roles of IFN-*γ* and TNF-*α*, our hypothesis that serum IFN-*γ* and TNF-*α* levels may be potential indicators for TAO disease severity or activity is thus substantiated.

Similar to IFN-*γ*, Th2 cytokine IL-4 stimulates retroorbital fibroblasts proliferation. Unlike IFN-*γ*, IL-4 stimulates the synthesis of type IV collagen instead of hyaluronic acid [37]. Moreover, IL-4 antagonizes IFN-*γ*-induced hyaluronic acid synthesis and may have some effect on the tissue repair process [37]. As shown in Tables 3 and 5, we found that serum IL-4 levels were significantly higher in inactive TAO than in active TAO and were negatively correlated with TRAb levels. This result is inconsistent with a recent study which reported no difference of serum IL-4 levels in GD patients with or without ophthalmopathy [21]. Possible explanations for this discrepancy might be the differences in the sample size or detection method. One study reported upregulation of serum IL-4 during successful treatment of TAO with corticosteroids [44]. Therefore, it is still plausible to consider serum IL-4 as an indicator for the inactive phase of TAO as well as for predicting therapeutic outcome. Further studies will be needed to clarify the roles of serum IL-4 in TAO.

The increased serum level of IL-17 in TAO patients and the correlation of IL-17 concentration with the clinical activity scores suggest that IL-17 may play a pathophysiological role in TAO development [25, 26]. However, although we found elevated serum IL-17 in GD, inactive TAO, and active TAO when compared to the normal control, there was no significant difference in IL-17 levels between inactive and active TAO. Additionally, the correlation of serum IL-17 in TAO with TRAb levels was not statistically significant. Since the role of IL-17 is still largely undefined, more evidence is needed to elucidate its role in TAO. As to the roles of chemokines in TAO, we found that levels of serum RANTES were not different among the groups; this finding is consistent with a previous study [45]. IP-10 is a member of CXC chemokine family and is also called CXCL10. IP-10 is induced by IFN-*γ* to recruit more Th1 T cells and to express CXCR3 (CXL10 receptor). We showed that serum IP-10 levels are higher in TAO than in the normal control group, but lower than in the GD only group. Although the levels of IP-10 in control group were higher than those in others' studies [31], which might be due to the differences in sample size or detection method, the elevation of IP-10 in TAO is in agreement with a previous report [38].

In summary, we found that serum levels of IFN-*γ* and TNF-*α* were positively correlated with TAO disease activity, while IL-4 levels were negatively correlated with TAO disease activity; here, TAO disease was indicated by TRAb levels. In conclusion, our findings suggest that serum levels of the Th1 cytokines IFN-*γ* and TNF-*α* may be useful as potential biomarkers for active TAO, while serum levels of the Th2 cytokine IL-4 may be useful as a potential biomarker for inactive TAO. Although pathogenic roles of IFN-*γ*, TNF-*α*, and IL-4 in the development of TAO are indicated by our studies, more investigation is needed to establish their specific contributions to TAO disease manifestation and activity.

## Figures and Tables

**Table 1:** Comparison of demographic data among study groups.

Group	Case	Age	Sex		Smoker	
			Male	Female	Yes	No
Normal	10	34.3 ± 12.2	5	5	3	7
GD	10	32.8 ± 10.7	5	5	3	7
Inactive TAO	20	38.3 ± 11.2	11	9	6	14
Active TAO	25	39.6 ± 15.1	12	13	11	14
*F*/*χ* ^2^		0.883	0.225		1.308	
*p*		0.455	0.974		0.727	

**Table 2:** Comparison of serum Th1 cytokine levels (pg/mL).

Group	Case	IFN-*γ*	TNF-*α*	IL-1*α*	IL-1Ra	IL-2
Normal	10	24.43 ± 19.77	7.43 ± 1.08	1.82 ± 0.11	14.07 ± 5.92	1.07 ± 0.27
GD	10	111.18 ± 117.69	13.67 ± 5.85^∗^	8.65 ± 1.26^▲^	22.40 ± 7.87	3.82 ± 0.31^▲^
Inactive TAO	20	70.77 ± 7.91^▲^	10.63 ± 3.99^∗^	7.92 ± 2.24^▲^	17.84 ± 13.18	0.84 ± 0.12^#^
Active TAO	25	88.04 ± 12.93^▲★^	15.73 ± 7.83^▲★^	9.25 ± 2.01^▲^	18.71 ± 12.74	0.77 ± 0.20^#^
*F*	—	30.290	14.034	242.21	0.894	279.10
*p*	—	0.000	0.000	0.000	0.450	0.000

^∗^ *p* < 0.05 versus normal; ^▲^ *p* < 0.001 versus normal; ^#^ *p* < 0.001 versus GD; ^★^ *p* < 0.05 versus inactive TAO.

**Table 3:** Comparison of serum Th2 cytokine levels (pg/mL).

Group	Case	IL-4	IL-6
Normal	10	0.75 ± 0.15	1.02 ± 0.16
GD	10	3.41 ± 0.53^▲^	18.72 ± 2.15^▲^
Inactive TAO	20	3.96 ± 1.51^▲^	8.10 ± 1.88^▲#^
Active TAO	25	2.04 ± 0.64^▲#★^	8.90 ± 1.08^▲#^
*F*	—	117.00	678.47
*p*	—	0.000	0.000

^▲^ *p* < 0.001 versus normal; ^#^ *p* < 0.001 versus GD; ^★^ *p* < 0.001 versus inactive TAO.

**Table 4:** Comparison of IL17, RANTES, and IP-10 serum levels (pg/mL).

Group	Case	IL-17	RANTES	IP-10
Normal	10	11.79 ± 5.66	8145.45 ± 656.53	404.29 ± 150.44
GD	10	118.66 ± 92.80^∗^	8103.04 ± 1254.84	1661.76 ± 599.48^▲^
Inactive TAO	20	40.91 ± 10.93^▲^	8224.30 ± 665.39	711.28 ± 406.11^∗#^
Active TAO	25	50.15 ± 11.52^▲^	8318.13 ± 776.13	794.75 ± 451.50^∗#^
*F*	—	66.206	0.212	17.131
*p*	—	0.000	0.887	0.000

^∗^ *p* < 0.05 versus normal; ^▲^ *p* < 0.001 versus normal; ^#^ *p* < 0.01 versus GD.

**Table 5:** Correlation between serum cytokines and TRAb.

Measures compared	Spearman *r*	*p*
IFN-*γ*	0.470	0.001
TNF-*α*	0.309	0.039
IL-1*α*	0.310	0.038
IL-1Ra	0.011	0.943
IL-2	−0.352	0.018
IL-4	−0.340	0.022
IL-6	0.332	0.026
IL-17	0.286	0.057
RANTES	0.084	0.582
IP-10	−0.093	0.545
